# Systemic Antifolate Chemotherapy Does Not Select for Fluconazole-Resistant *Candida*: A Multicenter Clinical Study

**DOI:** 10.3390/pathogens14060574

**Published:** 2025-06-07

**Authors:** Dawid Żyrek, Joanna Nowicka, Magdalena Pajączkowska, Mariola Paściak, Katarzyna Machnik, Tomasz Werner, Zygmunt Konieczny, Piotr Jędrzejczak, Dominika Raźniewska, Gabriela Fijałkowska, Michał Piątek, Barbara Radecka, Kinga Żyrek, Elżbieta Woźniak-Grygiel, Iwona Dzieńdziora-Urbińska

**Affiliations:** 1 Department of Histology, Institute of Medical Sciences, University of Opole, 45-052 Opole, Poland; ewozniakg@uni.opole.pl (E.W.-G.); iwona.dziendziora@uni.opole.pl (I.D.-U.); 2 Department of Microbiology, Faculty of Medicine, Wroclaw Medical University, 50-367 Wroclaw, Poland; 3 Hirszfeld Institute of Immunology and Experimental Therapy, Polish Academy of Sciences, Rudolfa Weigla 12, 53-114 Wroclaw, Poland; 4 Clinical Department of Pulmonology, Institute of Medical Sciences, University of Opole, 45-052 Opole, Poland; 5 Department of Pulmonology with a Chemotherapy Subunit, Hospital in Glucholazy, Lompy 3, 48-340 Glucholazy, Poland; 6 Department of Clinical Oncology, Lower Silesian Center of Oncology, Pulmonology and Hematology, Grabiszyńska 105, 53-439 Wroclaw, Poland; 7 Department of Oncology, Institute of Medical Sciences, University of Opole, 45-040 Opole, Poland; 8 Division of Physiotherapy, Faculty of Health Sciences, Wroclaw Medical University, 50-367 Wroclaw, Poland

**Keywords:** cross-resistance, drug-induced resistance, antifolates, pemetrexed, methotrexate, *Candida*, yeast

## Abstract

Previous studies have demonstrated that *Candida* spp. isolates exposed in vitro to the folic acid antagonist methotrexate may develop multidrug cross-resistance to azole antifungals. The aim of this study was to determine whether systemic therapy with antineoplastic antifolates—pemetrexed or methotrexate—constitutes a risk factor for colonization or infection with fluconazole-resistant yeasts. The study group comprised 44 cancer patients who received high-dose systemic antifolate therapy, while the control group consisted of 48 patients without prior exposure to either methotrexate or pemetrexed. Oral swabs and relevant clinical data were collected from all participants. In total, 109 fungal strains representing 13 species were isolated, identified, and subsequently tested for fluconazole susceptibility. Fluconazole-resistant isolates were identified in 4 out of 44 (9.1%) antifolate-treated patients and in 3 out of 48 (6.3%) control patients. Our findings suggest that, although this phenomenon occurs in vitro, systemic antineoplastic antifolate therapy does not induce azole resistance among endogenous yeast species in vivo.

## 1. Introduction

Methotrexate (MTX) and pemetrexed (PMX) are antifolate cytostatic agents widely used in the treatment of various malignancies. These compounds share structural similarities and exert their antineoplastic activity by disrupting folic acid metabolism [1,2]. While MTX primarily inhibits dihydrofolate reductase, PMX exerts broader cytotoxic activity by targeting additional enzymes (thymidylate synthase and glycinamide ribonucleotide formyltransferase), which contributes to its greater antineoplastic potency [3]. Low-dose MTX therapy (5–20 mg/week) is widely used in dermatology, rheumatology, and the treatment of autoimmune diseases, whereas high-dose regimens are reserved for specific hematological malignancies and selected solid tumors. PMX, on the other hand, is administered exclusively at high doses as a standard therapeutic option in the treatment of neoplasms such as lung adenocarcinoma and malignant pleural mesothelioma [1,3].

Oncology patients undergoing chemotherapy are often immunocompromised and therefore particularly vulnerable to various fungal infections [4]. In this population, the timely initiation of effective antifungal therapy may significantly affect clinical outcomes. Given these considerations, the potential interaction between cytostatic therapy and antifungal resistance is of particular clinical interest.

Previous studies have demonstrated that, under laboratory conditions, the exposure of *Candida* spp. to MTX may induce multidrug cross-resistance to azole antifungals, even in strains that have never been exposed to these compounds [5,6,7]. This phenomenon has been linked to MTX-induced overexpression of the CaMDR1 efflux pump gene, which enhances the active removal of xenobiotics, including azole compounds, from fungal cells [7,8]. While this phenomenon is well documented in vitro, its clinical relevance remains unclear. Only one observational study to date has reported a weak association between long-term, low-dose MTX therapy and the occurrence of azole-resistant fungal isolates [9].

Given the structural and functional similarities between MTX and PMX, as well as their in vitro fungistatic effects, it has been hypothesized that systemic antifolate therapy with either of these compounds may contribute to the selection of drug-resistant fungal strains in vivo [5,9,10]. However, this hypothesis has not yet been evaluated in a clinical setting involving oncology patients on high-dose systemic antifolate therapy.

Therefore, the aim of this study was to determine whether such treatment constitutes a risk factor for the emergence of fluconazole-resistant endogenous yeast strains in the oral cavity of oncology patients.

## 2. Materials and Methods

Participants who consented to take part in the study were recruited from four healthcare centers in southern Poland: the Department of Clinical Oncology of the Lower Silesian Center of Oncology, Hematology, and Pulmonology in Wroclaw; the Clinical Department of Pulmonology at the Provincial Hospital in Opole; the Oncology Clinic with Daily Ward at the Opole Oncology Center; and the Department of Pulmonology with a Chemotherapy Subunit at the Hospital in Glucholazy. The study group consisted of 43 cancer patients treated with PMX and one patient who received systemic MTX therapy. In all cases, at least four weeks had elapsed between the first administration of the cytostatic agent and the collection of study material. The control group included 48 cancer patients who had never been exposed to either MTX or PMX. Oral swabs were collected from all participants. Six individuals originally enrolled in the control group later received PMX therapy, while one person from the control group was subsequently treated with MTX. In these cases, follow-up oral swabs were collected at least four weeks after the first administration of the respective antifolate. During the qualification process, the following clinical data were also collected: sex, age, indication for antifolate therapy, cumulative dose of an antifolate, comorbidities, current hospitalization status (outpatient or inpatient), prior hospitalization in the past three months and information on concomitant medications—particularly prior exposure to azole antifungals during the last three months. The most relevant clinical characteristics of both groups are summarized in Table 1.

The collected material was cultured in liquid medium and on Sabouraud agar (Biomaxima, Lublin, Poland) at 37 °C for 48 h under aerobic conditions. Isolated strains were identified using matrix-assisted laser desorption/ionization time-of-flight mass spectrometry (MALDI-TOF MS). MALDI-TOF MS analysis was performed using the Ultraflextreme mass spectrometer from Bruker Daltonics (Bremen, Germany) with Biotyper 3.1 software. The ethanol–formic acid extraction method, as recommended by the manufacturer, was utilized for isolate identification [11]. The spectra were externally calibrated using the E. coli DH5-alpha standard provided by Bruker Daltonics (Bremen, Germany).

For all strains, the minimum inhibitory concentration (MIC) for fluconazole was determined using the broth microdilution method in RPMI 1640 medium, in accordance with EUCAST guidelines [12]. Suspensions of the tested strains (0.5–2.5 × 10^3^ CFU per mL) were applied to 96-well polystyrene plates with previously prepared serial dilutions of fluconazole in concentrations ranging from 0.125 µg/mL to 256 µg/mL. The plates were incubated for 24 h at 37 °C. To determine MICs, the optical density (OD) after 24 h was read spectrophotometrically (BiochromAsys UVM 340) at a wavelength of 530 nm. All experiments included a strain growth control (positive control; K+) and a negative control (K-), which served as a medium sterility test. The MIC was considered to be the concentration of fluconazole at which the growth inhibition of at least 50% of microorganisms (using the equation (OD_well_ − OD_K-_)/(OD_K+_ − OD_K-_) ×100%) was detected. The data were interpreted based on the clinical breakpoints recommended by EUCAST [12].

Clinical and microbiological data obtained in both the study and control groups were compared and subjected to statistical analysis. Statistical analysis was performed using TIBCO Statistica v13.3 software (TIBCO Software Inc., Palo Alto, CA, USA). The Shapiro–Wilk test was used to assess the normality of continuous variables. Categorical variables were compared using Fisher’s exact test. Continuous variables were compared between the study and control groups using the Mann–Whitney U test, treating groups as independent. To avoid duplication bias, patients contributing samples to both groups (n = 7) were included only once, in the control group. For analyses of paired samples (before and after antifolate exposure), only patients from the control group who later received antifolate therapy were included. Patients with negative oral cultures either before or after antifolate therapy were excluded from MIC comparisons. In the paired sample analysis, fluconazole MIC values before and after antifolate exposure were compared using the Wilcoxon signed-rank test. Changes in the presence of azole-resistant strains were evaluated using McNemar’s test. A *p*-value ≤ 0.05 was considered statistically significant.

The study was conducted in accordance with the Declaration of Helsinki, and approved by the Bioethical Committee at Opole University, Poland (approval No. UO/0025/KB/2023 provided on 26 October 2023).

## 3. Results

Oral swab cultures from study participants yielded between one and three fungal isolates per individual, representing a total of 13 different species. Among patients in the study group, a total of 52 distinct fungal isolates were identified, while 57 distinct isolates were identified among patients in the control group. Table 2 provides a comparative overview of species distribution and fluconazole susceptibility profiles, including MIC values, for both groups.

No significant difference (Fisher’s exact test, *p* = 0.45; OR = 1.50) was observed in the frequency of fluconazole-resistant strains between the study and control groups. Both groups contained one resistant *C. albicans* strain (MIC = 8 µg/mL) and two intrinsically fluconazole-resistant *P. kudriavzevii* strains (MIC = 16 µg/mL). Additionally, *P. cactophila*, a species also intrinsically resistant to fluconazole, was detected exclusively in the study group. All resistant isolates originated exclusively from patients with lung cancer who were hospitalized at the time of swab collection and had also been hospitalized at least once within the three months preceding study enrollment. None of the patients from whom resistant strains were isolated had a history of prior antifungal therapy, including azole exposure.

**Figure 1:**
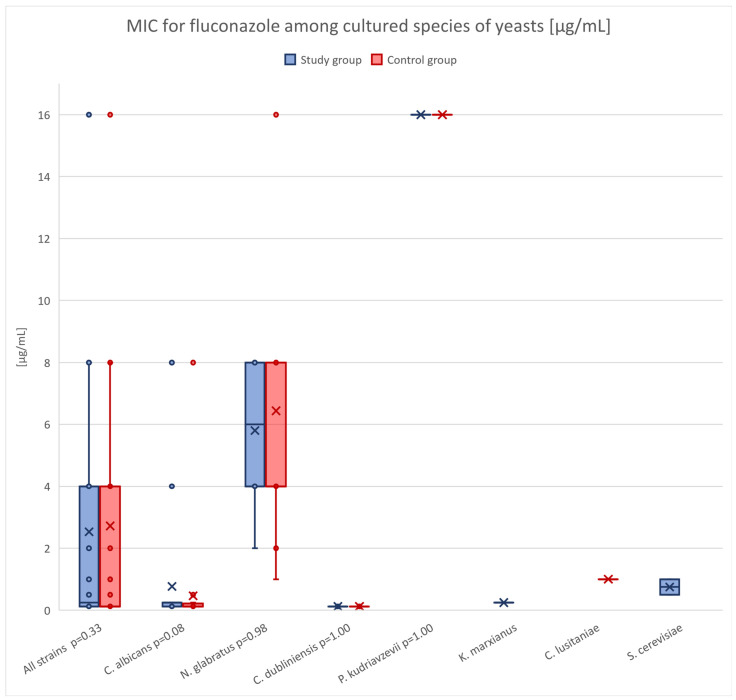
Distribution of fluconazole MIC values for yeast species isolated from patients in the control and study groups. Boxplots represent the interquartile range (IQR), with whiskers indicating the minimum and maximum non-outlier values. Individual data points represent MIC values for each isolate, while “×” marks indicate the mean MIC value for each species–group combination. The *p*-value shown next to each species identifier corresponds to the result of the Mann–Whitney U test comparing MIC values between the two groups.

Figure 1 presents fluconazole susceptibility profiles of fungal isolates from the study and control groups, organized by species. Although fluconazole MIC values tended to be higher among antifolate-treated patients—considering all strains (median MIC 0.250 vs. 0.125), *C. albicans* (median MIC 0.250 vs. 0.125), and *N. glabratus* (median MIC 6.000 vs. 4.000)—these differences were not statistically significant compared to those observed in patients not exposed to antifolates.

Oral swab samples obtained from patients both before and during antifolate therapy revealed no evidence of decreased fluconazole susceptibility among previously present strains (Wilcoxon signed-rank test, *p* = 1.00) or increased frequency of colonization with new resistant ones (McNemar’s test, *p* = 1.00). In two individuals (Patient 21 and Patient 23) who were colonized by *P. kudriavzevii* (a species intrinsically resistant to azoles) prior to the initiation of PMX therapy, this species was not detected in oral samples obtained following antifolate treatment. In contrast, following the initiation of PMX therapy, colonization in one patient (Patient 15) shifted from a fluconazole-susceptible *N. glabratus* strain to *P. cactophila*, a species intrinsically resistant to fluconazole (Table 3).

Detailed MIC data for individual isolates, along with basic clinical characteristics of the patients from whom they were obtained, are provided in the Supplementary Materials (Table S1).

## 4. Discussion

The literature on the induction of azole resistance in yeast exposed to antifolates under in vitro conditions is extensive and well documented [5,6,7,8,13]. The potential in vivo occurrence of this effect was suggested by a 2021 study that assessed azole-resistant *Candida* colonization in patients with rheumatic diseases undergoing long-term, low-dose MTX therapy [9]. Although the difference did not reach statistical significance in that study, resistant isolates were observed nearly twice as frequently in MTX-treated patients compared to antifolate-naive individuals. Notably, all resistant strains were isolated from patients taking MTX at the higher end of the dosing range (20–25 mg/week), suggesting a potential dose-dependent effect.

In light of these findings, the present study was designed to investigate whether high-dose systemic antifolate therapy produces comparable in vivo effects in oncology patients. The working hypothesis proposed that antifolate therapy, particularly with high cumulative doses and prolonged treatment duration, would exert sufficient selective pressure to increase the frequency of azole-resistant strains, either by promoting the expansion of intrinsically resistant species such as *P. kudriavzevii* or by inducing acquired resistance in previously susceptible strains.

Regarding fluconazole susceptibility, although median MIC values were slightly higher in antifolate-treated patients—both overall (0.250 vs. 0.125 µg/mL) and within *N. glabratus* (6.00 vs. 4.00 µg/mL) and *C. albicans* (0.250 vs. 0.125 µg/mL) isolates—the differences were not statistically significant. Furthermore, in patients who underwent oral swab collection both before and after antifolate therapy initiation, no evidence of decreased fluconazole susceptibility or replacement by azole-resistant strains was observed.

Resistant fungal isolates were similarly distributed across both groups. All fluconazole-resistant isolates were identified exclusively in patients who were hospitalized at the time of sampling and had experienced at least one prior hospitalization within the previous three months. This aligns with existing evidence identifying hospitalization as a major risk factor for colonization or infection with resistant fungal strains [14]. Other recognized risk factors (such as chronic kidney disease, neutropenia, broad-spectrum antibiotic use, previous antifungal treatment and solid organ transplantation) were not present among patients from whom resistant strains were isolated in this study [4,15,16]. Notably, at the time of sampling, none of the patients included in the study had an active fungal infection, were receiving antifungal treatment, or had undergone mycological diagnostics for any other reason. Among those who had received azole treatment within the three months preceding oral swab collection, two presented with symptomatic oral candidiasis and one was treated for vulvovaginal candidiasis. In all cases, fluconazole was prescribed empirically, without prior mycological confirmation of fungal etiology.

Overall, these results suggest that the selective pressure exerted by antifolates on the oral mycobiome is insufficient to drive significant shifts in antifungal resistance patterns. The potential for resistance induction observed in vitro may not translate into clinically relevant effects under standard therapeutic conditions. Alternatively, the frequency or magnitude of this effect may be low, and the sample size of our study was insufficient to detect it. Another possible explanation for the discrepancy between in vitro responses and clinical observations lies in the differential distribution of antifolates across various tissues and bodily fluids, combined with other pharmacokinetic factors such as intracellular metabolism. These aspects may influence the actual concentrations of antifolates encountered by fungal strains colonizing specific anatomical niches, such as the oral mucosa. The intravenous administration of MTX at 1 mg/kg yields an average salivary concentration of 130 ± 30 ng/mL, which represents only a small percentage of its plasma concentration [17]. PMX has not been specifically studied in this context; however, based on its pharmacokinetic profile—particularly strong plasma protein binding—it is reasonable to assume that its salivary penetration is similarly limited [2,3]. In contrast, laboratory protocols for inducing azole resistance typically involve at least 24 h of exposure to antifolate concentrations equal to or exceeding peak therapeutic plasma levels [5,6,13]. Such conditions are likely to exert stronger selective pressure and may trigger mechanisms involved in the active efflux of xenobiotics from fungal cells. The examination of samples from different body sites, such as the skin, blood or catheterized areas, may offer new insights, as antifolate distribution might modulate fungal colonization patterns differently depending on anatomical location.

Interestingly, antifolate-treated patients were more likely to have negative fungal cultures from oral swabs compared to antifolate-naive individuals, although the difference was not statistically significant (OR 1.62, *p* = 0.41). Among PMX-treated individuals with negative cultures, the average cumulative PMX dose was nearly double that of patients with positive cultures (8035 ± 3365 mg vs. 4000 ± 2630 mg, *p* = 0.006). These findings may reflect the fungistatic properties of antifolates observed under laboratory conditions [10]. From a clinical perspective, the suppression or eradication of intrinsically resistant species such as *P. kudriavzevii* could represent a potentially beneficial effect, reducing the risk of fungal overgrowth in immunocompromised patients. Moreover, antifolates, when combined with azoles, exert a synergistic fungistatic effect on the filamentous form of *Candida*, which paradoxically could suggest that azoles may be the preferred antifungal agents for patients undergoing systemic antifolate therapy who develop fungal infections [10,18]! Given the potential clinical significance of this effect, further studies are required to investigate this phenomenon in greater detail.

Nevertheless, in light of all the above-mentioned observations, there is currently no compelling reason to modify existing clinical strategies for patients receiving systemic antifolate therapy. Azoles may be considered an appropriate empirical option for antifungal prophylaxis and treatment in an oncology setting.

### Limitations

This study has several limitations. Firstly, despite the involvement of multiple recruiting centers, the number of patients who met the inclusion criteria for the antifolate-treated group was lower than initially expected. As a result, the statistical power of the study to detect subtle differences between groups was limited. Since our results suggest a trend towards a higher prevalence of resistant fungal isolates among antifolate-treated patients, it is possible that this association would have reached statistical significance in a larger sample.

A further limitation is that only one patient in our cohort received high-dose systemic MTX, significantly limiting our ability to evaluate the specific impact of this compound. Notably, this individual was initially assigned to the control group but was later switched to MTX therapy after standard treatment options had been exhausted. To better assess the clinical effects of MTX, future studies should include populations in which high-dose MTX remains a part of standard care, such as patients with lymphoproliferative or hematologic malignancies.

Lastly, due to the small number of patients sampled both before and after antifolate therapy, we were unable to fully distinguish between the acquisition of intrinsically resistant strains (for example multidrug-resistant hospital strains) and the emergence of resistance in previously susceptible endogenous flora. This question warrants further investigation in larger, longitudinal studies involving repeated sampling. Moreover, the authors believe that the routine, widespread implementation of mycological screening before and after immunosuppressive cancer therapy may help in tracking resistance emergence and optimizing antifungal management.

## 5. Conclusions

Systemic antifolate therapy was not associated with a statistically significant increase in the occurrence of fluconazole-resistant fungal strains in the oral cavity. While antifolate-induced cross-resistance has been observed in vitro, our findings do not support the presence of this phenomenon in clinical settings under the current treatment protocols.

## Figures and Tables

**Table 1:** Characteristics of the study participants. The first column presents *p*-values from the Mann–Whitney U test (for age) and Fisher’s exact test (for all other variables) comparing demographic and clinical characteristics between the study and control groups. To avoid duplication bias, patients contributing samples to both groups during statistical analysis were included only once, in the control group. No statistically significant differences were observed. SD—standard deviation; AC—adenocarcinoma; SCC—squamous cell carcinoma; SCLC—small cell lung cancer; NSCLC—non-small cell lung cancer.

	Study Group	Control Group
Age ± SD, (median) [years] *p* = 0.71	67.8 ± 6.7, (69)	67.5 ± 9.9, (68.5)
Gender *p* = 0.27	Male: 24/44 (54.6%) Female: 20/44 (45.5%)	Male: 30/48 (62.5%) Female: 18/48 (37.5%)
Cumulative Pemetrexed dose ± SD, (median) [mg]	4844.8 ± 4490.3, (2960)	-
Cumulative Methotrexate dose ± SD, (median) [mg]	120 ± 0, (0)	-
Azole intake in the last 3 months (% of participants) *p* = 0.63	1/44 (2.3%)	2/48 (4.2%)
Hospitalization status (% of participants) *p* = 0.25	Outpatient: 9/44 (20.5%) Inpatient: 35/44 (79.6%)	Outpatient: 6/48 (12.5%) Inpatient: 42/48 (87.5%)
Prior hospitalization in the last 3 months (% of participants) *p* = 0.10	35/44 (79.6%)	42/48 (64.6%)
Type of neoplasm	Lung cancer: 40/44 (90.9%) - AC: 40/44	Lung cancer: 36/48 (75.0%) - AC: 16/48 - SCC: 12/48 - SCLC: 4/48 - NSCLC, nonspecified: 4/48
	Pleural mesothelioma: 3/44 (6.8%)	Pleural mesothelioma: 1/48 (2.1%)
	Urinary bladder cancer: 1/44 (2.3%)	Urinary bladder cancer: 1/48 (2.1%)
		Gastric cancer: 2/48 (4.2%) Colorectal cancer: 4/48 (8.3%) Cholangiocarcinoma: 1/48 (2.1%) Pancreatic cancer: 1/48 (2.1%) Pharyngeal cancer: 1/48 (2.1%) Prostate cancer: 1/48 (2.1%)

**Table 2:** Characteristics of fungal strains cultured from oral swab samples collected from patients in the study and control groups. MIC—minimum inhibitory concentration; IQR—interquartile range.

	Study Group	Control Group
Culture-positive samples	34/44 (77.3%)	41/48 (85.4%)
Total number of strains	52	56
Average number of strains per positive sample ± SD	1.5 ± 0.6	1.4 ± 0.5
Fluconazole-resistant isolates (% of samples)	4/44 (9.1%)	3/48 (6.3%)
Average MIC ± SD [µg/mL]	2.235 ± 3.921	2.674 ± 4.526
Number of strains (% of samples containing the species)	*- Nakaseomyces glabratus* (formerly *Candida glabrata*)—10 (22.7%) *- Candida albicans*—25 (47.7%) - *Candida dubliniensis*—4 (9.1%) *- Candida tropicalis*—4 (4.5%) *- Pichia kudriavzevii* (formerly *Candida krusei*)—2 (4.5%) *- Pichia cactophila*—1 (2.3%) *- Kluyveromyces marxianus* (formerly *Candida kefyr*)—1 (2.3%) *- Kluyveromyces lactis*—1 (2.3%) *- Saccharomyces cerevisiae*—2 (4.5%) *- Hyphopichia burtonii*—1 (2.3%) *- Hanseniaspora uvarum*—1 (2.3%)	*- Nakaseomyces glabratus* (formerly *Candida glabrata*)—16 (31.3%) *- Candida albicans*—27 (56.3%) *- Candida dubliniensis*—9 (16.7%) *- Pichia kudriavzevii* (formerly *Candida krusei*)—2 (4.2%) *- Kluyveromyces marxianus* (formerly *Candida kefyr*)—1 (2.1%) *- Clavispora lusitaniae* (formerly *Candida lusitaniae*)—1 (2.1%) *- Torulaspora delbrueckii*—1 (2.1%)

**Table 3:** Summary of fungal strains cultured from oral swab samples collected from patients both before and after the initiation of antifolate therapy. Fluconazole-resistant species are marked with an asterisk (*). MIC—minimum inhibitory concentration for fluconazole; MTX—methotrexate; PMX—pemetrexed.

	Before Antifolate	Cumulative Dose	After Antifolate
Patient 15	*N. glabratus*, MIC = 16.000 µg/mL	905 mg of PMX	P. cactophila *
Patient 18	*C. albicans*, MIC = 0.125 µg/mL *C. albicans*, MIC = 0.250 µg/mL	11,000 mg of PMX	*C. albicans*, MIC = 0.125 µg/mL
Patient 19	NEGATIVE CULTURE	3920 mg of PMX	*C. albicans*, MIC = 0.125 µg/mL *H. uvarum*
Patient 21	P. kudriavzevii *, MIC = 16.000 µg/mL *C. albicans*, MIC = 0.125 µg/mL	3240 mg of PMX	*C. albicans*, MIC = 0.125 µg/mL *C. albicans*, MIC = 0.125 µg/mL
Patient 22	*N. glabratus*, MIC = 1.000 µg/mL *N. glabratus*, MIC = 4.000 µg/mL	3360 mg of PMX	NEGATIVE CULTURE
Patient 23	*C. albicans*, MIC = 0.125 µg/mL P. kudriavzevii *, MIC = 16.000 µg/mL	2340 mg of PMX	*C. albicans*, MIC = 0.125 µg/mL *C. dubliniensis*, MIC = 0.125 µg/mL
Patient 28	NEGATIVE CULTURE	120 mg of MTX	NEGATIVE CULTURE

## Data Availability

All relevant data is included in the article and Supplementary Materials. Any additional information may be provided upon written request sent to dawid.zyrek@uni.opole.pl.

## Supplementary Materials

The following supporting information can be downloaded at https://www.mdpi.com/article/10.3390/pathogens14060574/s1, Table S1: Characteristics of fungal strains and study participants.

## Abbreviations

The following abbreviations are used in this manuscript:

MTX	Methotrexate
PMX	Pemetrexed
FLU	Fluconazole
MIC	Minimum inhibitory concentrations
CLSI	Clinical and Laboratory Standards Institute
OD	Optical density
K-	Negative control
K+	Positive control
SDD	Susceptible-dose dependent
SD	Standard deviation
AC	Adenocarcinoma
SCC	Squamous cell carcinoma
SCLC	Small cell lung cancer
NSCLC	Non-small cell lung cancer; unspecified
