# Supplementary material for: Systemic Antifolate Chemotherapy Does Not Select for Fluconazole-Resistant Candida: A Multicenter Clinical Study
Source: Pathogens. 2025 Jun 7;14(6):574. doi: 10.3390/pathogens14060574 (PMC12195857; doi:10.3390/pathogens14060574)
Supplement: Supplementary file 1 [file pathogens-14-00574-s001.zip › pathogens-3656682-supplementary.pdf]

Sample number	Strain species number 1	MIC for Fluconazole - Strain number 1	Strain species number 2	MIC for Fluconazole - Strain number 2	Strain species number 3	MIC for Fluconazole - Strain number 3	Strains per sample	Control group(C)/ Study group (S)	Cumulative dose	Cause of treatment	Age	Sex (F- female, M- male)	Azole intake in the last 3 months (0- no; 1-yes)	Outpatient (0)/ Inpatient (1)	Prior hospitalization in the last 3 months (0- no; 1-yes)
1	*Hyphopichia burtonii*	-					1	S	2960 PMX	LUNG CANCER- ADENOCARCINOMA	72	F	0	0	0
2	*Candida albicans*	0,250					1	C	0	LUNG CANCER- ADENOCARCINOMA	68	M	0	1	0
3	*Candida dubliniensis*	0,125					1	S	20000 PMX	LUNG CANCER- ADENOCARCINOMA	76	M	0	1	1
4	*Candida albicans*	0,125					1	C	0	NON-SMALL CELL LUNG CANCER; NONSPECIFIED	70	F	0	1	1
5	*Candida dubliniensis*	0,125	*Nakaseomyces glabratus*	4,000			2	C	0	NON-SMALL CELL LUNG CANCER; NONSPECIFIED	57	M	0	0	0
6	*Candida dubliniensis*	0,125	*Nakaseomyces glabratus*	4,000	*Sacharomyces cerevisiae*	0,500	3	S	1840 PMX	LUNG CANCER- ADENOCARCINOMA	56	F	0	1	1
7	*negative culture*						0	S	8500 PMX	LUNG CANCER- ADENOCARCINOMA	71	M	0	1	0
8	*negative culture*						0	S	8500 PMX	LUNG CANCER- ADENOCARCINOMA	71	M	0	1	0
9	*Candida tropicalis*	0,500	*Candida tropicalis*	0,250			2	S	6760 PMX	LUNG CANCER- ADENOCARCINOMA	70	F	0	1	1
10	*negative culture*						0	S	8500 PMX	LUNG CANCER- ADENOCARCINOMA	71	M	0	1	1
11	*Nakaseomyces glabratus*	16,000					1	C	0	LUNG CANCER- ADENOCARCINOMA	67	M	0	1	1
12	*Nakaseomyces glabratus*	2,000	*Candida albicans*	0,125			2	S	1620 PMX	LUNG CANCER- ADENOCARCINOMA	70	F	1	1	1

13	*Candida albicans*	8,000	*Nakaseomyces glabratus*	4,000			2	S	2460 PMX	LUNG CANCER-ADENOCARCINOMA	78	F	0	1	1
14	*Candida albicans*	4,000					1	S	3380 PMX	LUNG CANCER-ADENOCARCINOMA	68	M	0	1	1
15A	*Nakaseomyces glabratus*	16,000					1	C	0	LUNG CANCER-ADENOCARCINOMA	64	M	0	1	0
15B	*Pichia cactophila*	-					1	S	905 PMX	LUNG CANCER-ADENOCARCINOMA	64	M	0	1	1
16	*Kluyveromyces marxianus*	0,250	*Candida albicans*	0,125			2	S	2560 PMX	LUNG CANCER-ADENOCARCINOMA	71	F	0	1	1
17	*Candida albicans*	0,125					1	S	5400 PMX	LUNG CANCER-ADENOCARCINOMA	63	M	0	1	1
18A	*Candida albicans*	0,125	*Candida albicans*	0,250			2	C	0	LUNG CANCER-ADENOCARCINOMA	55	M	0	1	1
18B	*Candida albicans*	0,125					1	S	11000 PMX	LUNG CANCER-ADENOCARCINOMA	55	M	0	1	1
19	*negative culture*						0	C	0	LUNG CANCER-ADENOCARCINOMA	67	M	0	1	1
20	*Candida albicans*	0,125	*Hanseniaspora uvarum*	-			2	S	3920 PMX	LUNG CANCER-ADENOCARCINOMA	68	M	0	1	1
21A	*Pichia kudriavzevii*	16,000	*Candida albicans*	0,125			2	C	0	LUNG CANCER-ADENOCARCINOMA	56	M	0	1	1
21B	*Candida albicans*	0,125	*Candida albicans*	0,125			2	S	3240 PMX	LUNG CANCER-ADENOCARCINOMA	56	M	0	1	1
22A	*Nakaseomyces glabratus*	1,000	*Nakaseomyces glabratus*	4,000			2	C	0	LUNG CANCER-ADENOCARCINOMA	73	F	0	1	1
22B	*negative culture*						0	S	3360 PMX	LUNG CANCER-ADENOCARCINOMA	74	F	0	1	1
23A	*Candida albicans*	0,125	*Pichia kudriavzevii*	16,000			2	C	0	LUNG CANCER-ADENOCARCINOMA	60	M	0	1	1
23B	*Candida dubliniensis*	0,125	*Candida albicans*	0,125			2	S	2340 PMX	LUNG CANCER-ADENOCARCINOMA	60	M	0	1	1
24	*Pichia kudriavzevii*	16,000					1	S	12800 PMX	LUNG CANCER-ADENOCARCINOMA	61	F	0	1	1
25	*Candida albicans*	0,125					1	C	0	LUNG CANCER-ADENOCARCINOMA	69	F	1	1	1
26	*Sacharomyces cerevisiae*	1,000					1	S	900 PMX	LUNG CANCER-ADENOCARCINOMA	80	M	0	1	1

27	*Candida albicans*	0,125					1	C	0	LUNG CANCER-ADENOCARCINOMA	81	M	0	1	1
28A	*negative culture*							C	0	URINARY BLADDER CANCER	69	M	0	0	1
28B	*negative culture*							S	120 MTX	URINARY BLADDER CANCER	69	M	0	0	1
29	*Candida albicans*	0,125					1	C	0	LUNG CANCER-ADENOCARCINOMA	81	M	0	1	1
30	*Pichia kudriavzevii*	16,000	*Nakaseomyces glabratus*	4,000			2	S	1530 PMX	LUNG CANCER-ADENOCARCINOMA	62	F	0	1	1
31	*Candida tropicalis*	0,125	*Candida tropicalis*	0,125			2	S	420 PMX	LUNG CANCER-ADENOCARCINOMA	70	F	0	1	1
32	*Candida albicans*	0,125	*Nakaseomyces glabratus*	4,000			2	C	0	LUNG CANCER-ADENOCARCINOMA	77	M	0	1	1
33	*Candida albicans*	0,125					1	C	0	LUNG CANCER-ADENOCARCINOMA	63	M	0	1	1
34	*Candida albicans*	0,125	*Candida albicans*	0,250			2	S	5700 PMX	LUNG CANCER-ADENOCARCINOMA	66	M	0	1	1
35	*Kluyveromyces lactis*	0,250					1	S	1400 PMX	LUNG CANCER-ADENOCARCINOMA	80	F	0	1	1
36	*negative culture*						0	S	9855 PMX	LUNG CANCER-ADENOCARCINOMA	69	M	0	0	0
37	*Candida albicans*	0,250	*Nakaseomyces glabratus*	8,000			2	S	5000 PMX	LUNG CANCER-ADENOCARCINOMA	59	M	0	1	1
38	*Candida albicans*	0,250	*Nakaseomyces glabratus*	8,000			2	S	3780 PMX	PLEURAL MESOTHELIOMA	53	M	0	1	1
39	*Candida albicans*	0,250					1	S	2700 PMX	LUNG CANCER-ADENOCARCINOMA	71	M	0	1	1
40	*Candida albicans*	0,250					1	S	1810 PMX	PLEURAL MESOTHELIOMA	76	F	0	0	0
41	*Candida albicans*	0,250					1	S	2730 PMX	LUNG CANCER-ADENOCARCINOMA	69	F	0	0	1
42	*Candida albicans*	0,250					1	S	1400 PMX	LUNG CANCER-ADENOCARCINOMA	66	F	0	1	1
43	*Candida albicans*	0,125	*Candida albicans*	0,125	*Candida albicans*	0,125	3	S	2975 PMX	LUNG CANCER-ADENOCARCINOMA	64	M	0	1	1
44	*Candida albicans*	0,125					1	S	1800 PMX	LUNG CANCER-ADENOCARCINOMA	59	M	0	1	1

45	*Candida albicans*	0,250	*Nakaseomyces glabratus*	8,000			2	S	1300 PMX	LUNG CANCER-ADENOCARCINOMA	78	F	0	1	1
46	*negative culture*						0	S	1700 PMX	LUNG CANCER-ADENOCARCINOMA	65	F	0	1	1
47	*negative culture*						0	S	9000 PMX	LUNG CANCER-ADENOCARCINOMA	71	F	0	1	1
48	*Nakaseomyces glabratus*	4,000					1	S	1380 PMX	LUNG CANCER-ADENOCARCINOMA	74	F	0	1	1
49	*Candida albicans*	0,125					1	C	0	LUNG CANCER-SQUAMOUS CELL CARCINOMA	63	F	0	0	0
50	*Candida albicans*	0,250	*Nakaseomyces glabratus*	8,000			2	S	1000 PMX	LUNG CANCER-ADENOCARCINOMA	63	F	0	1	1
51	*Candida dubliniensis*	0,125	*Nakaseomyces glabratus*	8,000			2	C	0	SMALL CELL LUNG CANCER	64	F	0	1	1
52	*Candida albicans*	0,125	*Nakaseomyces glabratus*	8,000			2	C	0	LUNG CANCER-SQUAMOUS CELL CARCINOMA	69	M	0	1	1
53	*negative culture*						0	C	0	LUNG CANCER-SQUAMOUS CELL CARCINOMA	80	M	0	0	0
54	*Candida albicans*	8,000					1	C	0	LUNG CANCER-SQUAMOUS CELL CARCINOMA	65	M	0	1	1
55	*negative culture*						0	C	0	PHARYNGEAL SQUAMOUS CELL CARCINOMA	69	M	0	1	1
56	*Candida albicans*	0,125					1	C	0	PROSTATE ADENOCARCINOMA	72	M	0	1	1
57	*Candida albicans*	0,125					1	C	0	LUNG CANCER-SQUAMOUS CELL CARCINOMA	66	M	0	1	1
58	*Nakaseomyces glabratus*	4,000					1	C	0	PANCREATIC ADENOCARCINOMA	75	M	0	1	0
59	*Candida albicans*	0,125	*Nakaseomyces glabratus*	2,000			2	C	0	NON-SMALL CELL LUNG CANCER; NONSPECIFIED	80	M	0	1	0
60	*Candida albicans*	0,250					0	C	0	LUNG CANCER-ADENOCARCINOMA	59	F	0	1	0

61	*Candida albicans*	0,250	*Nakaseomyces glabratus*	8,000			2	C	0	LUNG CANCER-SQUAMOUS CELL CARCINOMA	67	F	0	1	0
62	*Candida albicans*	0,500					1	C	0	COLORECTAL ADENOCARCINOMA	69	F	0	1	1
63	*Candida dubliniensis*	0,125					1	C	0	COLORECTAL ADENOCARCINOMA	72	M	0	1	1
64	*Candida albicans*	0,125					1	S	15200 PMX	LUNG CANCER-ADENOCARCINOMA	62	F	0	0	0
65	*Candida albicans*	0,125					1	C	0	SMALL CELL LUNG CANCER	68	M	0	1	0
66	*Torulaspora delbrueckii*	-					1	C	0	COLORECTAL ADENOCARCINOMA	45	F	0	1	1
67	*Nakaseomyces glabratus*	4,000	*Candida dubliniensis*	0,125			2	C	0	LUNG CANCER-SQUAMOUS CELL CARCINOMA	65	M	0	1	1
68	*Candida dubliniensis*	0,125					1	C	0	COLORECTAL ADENOCARCINOMA	73	F	0	1	1
69	*Candida dubliniensis*	0,125					1	C	0	GASTRIC ADENOCARCINOMA	26	F	0	1	1
70	*negative culture*						0	C	0	SMALL CELL LUNG CANCER	67	F	0	1	0
71	*Candida dubliniensis*	0,125					1	S	2850 PMX	LUNG CANCER-ADENOCARCINOMA	65	M	0	0	0
72	*Candida albicans*	0,125	*Nakaseomyces glabratus*	8,000			2	C	0	GASTRIC ADENOCARCINOMA	75	F	0	1	1
73	*Nakaseomyces glabratus*	8,000					1	S	950 PMX	LUNG CANCER-ADENOCARCINOMA	70	M	0	1	0
74	*Nakaseomyces glabratus*	4,000	*Candida albicans*	0,125			2	C	0	LUNG CANCER-ADENOCARCINOMA	86	M	0	1	1
75	*Candida dubliniensis*	0,125	*Candida dubliniensis*	0,125			2	C	0	PLEURAL MESOTHELIOMA	76	M	0	1	0
76	*Candida dubliniensis*	0,125					1	C	0	SMALL CELL LUNG CANCER	64	M	0	1	1
77	*Clavispora lusitaniae*	1,000					1	C	0	LUNG CANCER-SQUAMOUS CELL CARCINOMA	60	M	0	1	0

78	*negative culture*						0	C	0	CHOLANGIOCARCINOMA	76	M	1	1	1
79	*Candida albicans*	0,125					1	C	0	LUNG CANCER-ADENOCARCINOMA	64	F	0	1	0
80	*Candida albicans*	0,250	*Nakaseomyces glabratus*	4,000			2	C	0	LUNG CANCER-SQUAMOUS CELL CARCINOMA	69	F	0	0	0
81	*Candida albicans*	0,125					1	C	0	NON-SMALL CELL LUNG CANCER; NONSPECIFIED	73	F	0	0	0
82	*Nakaseomyces glabratus*	8,000	*Candida albicans*	0,250			2	C	0	LUNG CANCER-SQUAMOUS CELL CARCINOMA	69	F	0	1	1
83	*Kluyveromyces marxianus*	0,250					1	C	0	LUNG CANCER-SQUAMOUS CELL CARCINOMA	76	M	0	1	1
84	*negative culture*						0	S	11900 PMX	LUNG CANCER-ADENOCARCINOMA	73	M	0	0	0
85	*negative culture*						0	S	11000 PMX	PLEURAL MESOTHELIOMA	72	M	0	0	0
86	*negative culture*						0	C	0	LUNG CANCER-ADENOCARCINOMA	60	F	0	1	1

Table S1. Detailed MIC data for individual isolates, along with basic clinical characteristics of the patients from whom they were obtained. Samples obtained from the same patient both before and afer antifolate treatment have the same color; PMX- pemetrexed; MTX- methotrexate; MIC- minimum inhibitory concentration.
